# The therapeutic potential of natural products for pediatric psoriasis: targeting the immune microenvironment

**DOI:** 10.3389/fped.2026.1834627

**Published:** 2026-06-24

**Authors:** Zitong Wang, Jingyu Zhang, Suwen Liu, Hongxiang Chen

**Affiliations:** 1 Department of Dermatology, Zhongnan Hospital of Wuhan University, Wuhan University, Wuhan, China; 2 The Second Clinical College of Wuhan University, Wuhan, China; 3 Department of Dermatology, Union Hospital, Tongji Medical College, Huazhong University of Science and Technology, Wuhan, China; 4 Department of Dermatology, Shenzhen Nanshan People's Hospital/The 6th Affiliated Hospital of Shenzhen University Health Science Center, Shenzhen, China

**Keywords:** immune microenvironment, immunomodulation, natural products, pediatric psoriasis, therapeutic strategies

## Abstract

Psoriasis is a chronic, recurrent, inflammatory skin disorder closely associated with dysregulation of the immune microenvironment. Conventional systemic therapies often face limitations in children due to safety concerns, highlighting the urgent need for safer and effective treatment options. Natural products, characterized by their multi-target and multi-pathway actions along with relatively favorable safety profiles, as suggested primarily by adult and preclinical studies, have shown great potential in modulating the immune microenvironment. This review summarizes recent advances in the use of natural products—including plant extracts, active components, and compound formulations—in reshaping the immune microenvironment of psoriatic lesions by regulating T cell subsets, cytokine networks, innate immune cell functions, and skin barrier integrity. We explicitly distinguish between evidence derived from pediatric clinical studies, adult studies, and preclinical models. Furthermore, the article explores the implications of these findings for developing novel therapeutic strategies specifically tailored for pediatric psoriasis, addressing current challenges, and outlining future directions for translational research. By integrating these insights, this review aims to provide a comprehensive understanding of how natural products can contribute to safer and more effective immunomodulatory treatments in children with psoriasis.

## 1 Introduction

Psoriasis is a chronic inflammatory skin disorder characterized by aberrant keratinocyte proliferation and immune dysregulation, with a complex pathophysiology involving multiple cellular and molecular pathways (1, 2). One-third of psoriasis cases start during childhood (3). In pediatric populations, psoriasis presents unique challenges due to the ongoing growth and development of children, necessitating careful consideration of treatment safety, tolerability, and impact on quality of life (4). The immunopathogenesis of psoriasis has been recently reviewed in several publications (5, 6). No relevant differences appear to exist between the pathogenic mechanisms in adult and pediatric psoriasis. It is believed that an interaction among several immune cells, such as keratinocytes, dendritic cells (DCs), different T cell subsets including T helper 1 (Th1), Th17, and Th22, as well as T cytotoxic 17 (Tc17), neutrophils, and mast cells, together with the overexpression of several cytokines, such as interferon-alpha (IFN-*α*), IFN-γ, tumor necrosis factor-alpha (TNF-*α*), interferon-17 (IL-17), IL-20, and IL-22, are mainly responsible for the development and maintenance of psoriatic lesions (7). This shared pathogenesis forms the rationale for cautiously extrapolating findings from adult studies to pediatric contexts, while acknowledging the need for pediatric-specific validation. The immunopathogenesis of psoriasis centers on the abnormal activation of the immune system, particularly the Th17/IL-23 axis, which drives a cascade of inflammatory responses leading to keratinocyte hyperproliferation and disruption of skin barrier function (5, 8, 9). This immune-mediated inflammation is accompanied by excessive angiogenesis and infiltration of various immune cells, including T cells, macrophages, and DCs, contributing to the chronicity and recurrence of the disease (10, 11).

Considering that pediatric psoriasis can impose a significant burden on the overall physical, psychological and social health of the child, its treatment methods require special consideration (12). Current therapeutic strategies for pediatric psoriasis include topical agents, phototherapy, conventional systemic drugs, and biologic therapies targeting key cytokines such as IL-17 and IL-23 (4). While biologics have demonstrated significant efficacy and favorable safety profiles in children, their high cost and limited long-term safety data in this population pose barriers to widespread use. Moreover, the chronic nature of psoriasis requires sustained disease management, emphasizing the need for treatments that are not only effective but also safe for long-term administration in growing children (13, 14). Consequently, there is a pressing demand for alternative or adjunctive therapies that can modulate the immune microenvironment of psoriatic lesions with minimal adverse effects.

Natural products have emerged as promising candidates in this context due to their broad spectrum of bioactive compounds exhibiting anti-inflammatory, antioxidant, and immunomodulatory properties (15–17). However, it is crucial to note that “natural” does not automatically equate to “safe,” especially in pediatric populations where physiological and metabolic systems are still maturing. These products often exert pleiotropic effects by targeting multiple signaling pathways and cellular components involved in psoriasis pathogenesis. For instance, various plant-derived molecules have been shown *in vitro* and in animal models to inhibit pro-inflammatory cytokines, suppress keratinocyte proliferation, and restore immune homeostasis through modulation of pathways such as nuclear factor kappaB (NF-*κ*B), signal transducer and activator of transcription 3 (STAT3), and mitogen-activated protein kinases (MAPK) (18, 19). Additionally, natural products can influence the balance of immune cell subsets, including Th17 and regulatory T cells (Tregs), thereby reshaping the inflammatory milieu within psoriatic skin (20). These immunomodulatory properties have also been reviewed in the context of interleukin pathways (21).

Beyond terrestrial plants, marine microorganisms and edible fungi also represent important sources of bioactive natural compounds. Marine-derived polysaccharides and lipids have demonstrated preclinical anti-inflammatory effects by attenuating macrophage activation and cytokine production, suggesting their utility in managing chronic inflammatory skin diseases (22, 23). Similarly, edible mushrooms contain polysaccharides and terpenoids with immunoregulatory activities that may be harnessed for therapeutic purposes (24). Certain compounds also inhibit angiogenesis by targeting the focal adhesion kinase (FAK) and protein kinase B (Akt) pathways (25), while others modulate oxidative stress responses in DCs to restore the Th17/Tregs balance (26, 27). In the context of pediatric care, where the intestinal microbiota is still maturing, the ability of natural products to stabilize the skin-gut axis may offer a unique therapeutic advantage. These natural agents possess characteristics such as relatively low toxicity, multi-targeted mechanisms, and potential for oral administration, aligning well with the safety requirements for children (18).

At the molecular level, psoriasis involves dysregulated signaling pathways that promote keratinocyte hyperproliferation and inflammation. Key mediators include hypoxia-inducible factor-1*α* (HIF-1*α*), which enhances angiogenesis and inflammatory responses; STAT3, which drives keratinocyte proliferation and cytokine production; and phosphoinositide 3-kinase delta (PI3K*δ*), which sustains epithelial inflammation (28–30). Natural products have been reported to modulate these pathways effectively. For example, certain herbal extracts inhibit HIF-1*α*-mediated ferroptosis in keratinocytes, reducing hyperproliferation and inflammation (31). Others suppress STAT3 activation or PI3K*δ* signaling, thereby attenuating epidermal hyperplasia and immune cell infiltration (28, 29). These findings underscore the potential of natural products to intervene at critical nodes within the psoriasis immune network.

In pediatric psoriasis, where immune dysregulation is central to disease manifestation, the modulation of the immune microenvironment by natural products holds particular significance. The ability of these agents to target multiple immune pathways and cell types offers a strategic advantage in restoring immune balance and skin homeostasis. Moreover, their potentially favorable safety profiles and cost-effectiveness make them attractive candidates for long-term management in children. Integrating natural immunomodulators into therapeutic regimens could complement existing treatments, reduce reliance on biologics, and improve overall disease control and quality of life for children. However, these potential benefits must be weighed against significant gaps in pediatric-specific evidence, which are detailed throughout this review.

### 1.1 Literature search methodology

A narrative review of the literature was conducted to identify relevant studies on natural products for pediatric psoriasis. We systematically searched the PubMed, Scopus, and Web of Science databases for articles published up to December 2025. The search strategy combined terms related to “pediatric psoriasis,” “childhood psoriasis,” “natural products,” “phytochemicals,” “plant extracts,” “herbal medicine,” and “immunomodulation.” Studies were selected if they were peer-reviewed, published in English, and provided data on the immunomodulatory effects of natural products in psoriasis, with a preference for studies including pediatric populations. Due to the scarcity of pediatric-specific clinical trials, preclinical studies (*in vitro* and *in vivo*) and adult clinical studies were also included when they provided mechanistic insights or safety data relevant to pediatric applications. The distinction between these evidence sources is explicitly noted throughout the review.

This review aims to systematically elucidate the mechanisms by which natural products regulate the immune microenvironment in psoriasis, with a focus on their relevance to pediatric disease management. By highlighting key molecular targets and immunological pathways influenced by these products, we seek to provide a scientific basis for their incorporation into individualized, precision medicine approaches for children with psoriasis. Furthermore, we discuss the translational potential and clinical implications of natural immunomodulators, addressing challenges and future directions in their development as safe and effective therapies for pediatric psoriasis.

## 2 Modulation of key immune cells and molecular targets

### 2.1 Balancing adaptive immune cell subsets

The adaptive immune system plays a pivotal role in the pathogenesis of psoriasis, with dysregulated T cell subsets driving chronic inflammation and keratinocyte hyperproliferation. Among these, pathogenic Th17 cells are central effectors, producing pro-inflammatory cytokines such as IL-17A, IL-17F, and IL-22, which perpetuate the psoriatic inflammatory cascade (5). Natural products like triptolide glycosides from *Tripterygium wilfordii* and curcumin have demonstrated the capacity to inhibit Th17 differentiation and function by targeting key transcription factors STAT3 and MAPK, thereby reducing IL-17 family cytokine production and attenuating downstream inflammatory signaling, based on adult clinical trials and preclinical models (32, 33). This suppression of Th17 cells interrupts the IL-23/Th17 axis, a core pathogenic pathway in psoriasis. Concurrently, natural products such as quercetin and resveratrol have been shown to promote the expansion and functional enhancement of Tregs, which are crucial for maintaining immune tolerance and suppressing excessive inflammation (34). These products upregulate forkhead box protein 3 (Foxp3) expression and potentiate TGF-*β* signaling, either directly or indirectly via modulation of the gut microbiota, restoring the balance between effector and regulatory T cell populations. Moreover, certain phytochemicals exert broader immunomodulatory effects by rebalancing other T cell subsets implicated in psoriasis. For instance, inhibition of Th1 cells through downregulation of IFN-*γ* production, as well as modulation of other T cell subsets, contributes to correcting the skewed T cell subset distribution observed in psoriatic lesions (35). This multifaceted regulation of adaptive immunity by natural products not only dampens pathogenic inflammation but also fosters immune homeostasis, highlighting their therapeutic potential in psoriasis management.

### 2.2 Regulating cytokine and inflammatory networks

The inflammatory milieu in psoriasis is orchestrated by a complex network of cytokines and signaling pathways, with the IL-23/Th17 axis at its core. Natural products such as berberine and apigenin have been reported in preclinical studies to downregulate IL-23 production by DCs, thereby disrupting the initiation and maintenance of Th17-mediated inflammation (36, 37). Additionally, these agents can block the interaction between IL-17 and its receptor or inhibit downstream signaling cascades including NF-*κ*B and MAPK pathways, effectively severing the amplification loop of psoriatic inflammation. Beyond targeting IL-23/Th17, many bioactive compounds derived from natural products exhibit broad anti-inflammatory effects by suppressing key pro-inflammatory mediators such as TNF-α, IL-6, and IL-1β. Flavonoids like quercetin and luteolin inhibit the activation of the NOD-like receptor family pyrin domain containing 3 (NLRP3) inflammasome and modulate the NF-*κ*B axis or Notch, PI3K/Akt signaling, leading to decreased transcription of these cytokines and attenuation of inflammatory responses (38, 39). Furthermore, certain natural products enhance the expression of anti-inflammatory cytokines including IL-10 and IL-37, fostering an environment conducive to resolution of inflammation and tissue repair (31). This dual action—suppressing pro-inflammatory mediators while upregulating anti-inflammatory factors—enables natural products to recalibrate the immune microenvironment in psoriasis. Beyond inflammation, trace components of *Indigo naturalis* such as tryptanthrin intervene in the angiogenic cascade by inhibiting the phosphorylation of FAK and Akt pathways, thereby reducing vascular endothelial growth factor (VEGF) concentration and suppressing the formation of pathological vascular structures (22). Collectively, these immunomodulatory effects on cytokine networks and signaling pathways underscore the promise of natural products as adjunctive or alternative therapies for psoriasis, capable of targeting multiple nodes within the inflammatory cascade to achieve disease amelioration. Table 1 summarizes representative natural products and their multilevel immunomodulatory mechanisms in psoriasis.

**Table 1:** Representative natural products and their immunomodulatory mechanisms in psoriasis.

Natural Product	Source	Target Immune Cells	Key Molecular Targets	Effects	Evidence Level	Ref
Triptolide glycosides	*Tripterygium wilfordii*	Th17 cells	STAT3, ROR*γ*t	Inhibits Th17 differentiation; reduces IL-17 production	Adult Clinical Evidence	(33, 41)
Curcumin	*Curcuma longa*	Th17 cells, keratinocytes	STAT3, NF-*κ*B	Suppresses Th17 function; attenuates keratinocyte hyperproliferation	Adult Clinical Evidence	(32)
Resveratrol	Grapes, berries	Tregs, macrophages	SIRT1, NF-κB	Enhances Tregs function; promotes M2 macrophage polarization	Preclinical Evidence	(17, 19)
Berberine	*Coptis chinensis*	DCs, Th17 cells	IL-23, NF-κB	Downregulates IL-23; disrupts Th17 activation	Preclinical Evidence/Pilot Adult RCT	(36)
Apigenin	Parsley, celery	DCs	IL-23, NF-κB	Inhibits IL-23 production; reduces Th17-mediated inflammation	Preclinical Evidence	(37)
Quercetin	Onions, apples	Macrophages, neutrophils	NLRP3, MAPK	Inhibits inflammasome; reduces chemokine production	Preclinical Evidence	(39)
Glycyrrhizic acid	Licorice root	Keratinocytes, DCs	HMGB1, NF-κB	Reduces antimicrobial peptides; promotes tolerogenic DCs	Preclinical/Adult Clinical Pilot	(62, 65, 66)
Baicalin	*Scutellaria baicalensis*	Keratinocytes	CXCLs, S100 proteins	Suppresses IL-17-induced chemokine release	Preclinical Evidence	(42)
*Ganoderma lucidum* polysaccharides	Reishi mushroom	Macrophages, DCs	NF-κB, STAT3	Induce**s** M2 polarization; promotes tolerogenic DCs	Preclinical Evidence	(27)
Tryptanthrin	*Indigo naturalis*	Vascular endothelial cells	FAK, Akt, VEGF	Inhibits pathological angiogenesis	Preclinical/Case Reports	(22)
Oxymatrine	*Sophora flavescens*	Keratinocytes	p63	Stabilizes epidermal architecture without damaging basement membrane	Preclinical/Case Series	(43)

## 3 Regulation of skin innate immunity and barrier function

### 3.1 Modulating innate immune cells

The modulation of innate immune cell activity by natural products plays a pivotal role in managing inflammatory skin diseases such as pediatric psoriasis (40). Keratinocytes, the predominant cells in the epidermis, are not only structural components but also active participants in immune responses. Natural products like glycyrrhizic acid and baicalin have demonstrated the ability to directly suppress keratinocyte-mediated inflammatory responses (41, 42). Specifically, these agents inhibit the production of antimicrobial peptides such as LL-37, chemokines like C-X-C motif chemokine ligands (CXCLs), and S100 proteins induced by IL-17 stimulation, thereby reducing the recruitment and activation of immune cells at the lesion sites. This targeted suppression mitigates the amplification of local inflammation and immune cell infiltration, which are hallmarks of psoriatic pathology. In parallel, oxymatrine further stabilizes the epidermal architecture by inhibiting p63-mediated hyperproliferation and differentiation, crucially doing so without compromising the basement membrane's structural integrity (43). Beyond keratinocytes, DCs and macrophages are critical in antigen presentation and initiation of inflammatory cascades (44). Natural products such as ginsenosides and *Ganoderma lucidum* polysaccharides have been shown to promote the differentiation of DCs toward a tolerogenic phenotype, which dampens excessive immune activation (5, 44). Concurrently, these products facilitate the polarization of macrophages from the pro-inflammatory M1 phenotype to the anti-inflammatory M2 phenotype, thereby attenuating the inflammatory milieu. This dual modulation at the antigen-presenting and effector cell levels provides a comprehensive approach to controlling skin inflammation. Neutrophil infiltration is another key feature in psoriatic lesions, contributing to microabscess formation and tissue damage (45–47). Flavonoids, a class of natural polyphenols, have been reported to inhibit the production of neutrophil-attracting chemokines such as CXCL1 and CXCL8, effectively reducing neutrophil recruitment to the skin (48, 49). This action not only limits neutrophil-mediated tissue injury but also curtails the perpetuation of inflammation (18). Collectively, these natural products exert multi-level immunomodulatory effects by targeting keratinocytes, DCs, macrophages, and neutrophils, thereby restoring immune homeostasis in the skin. Their ability to interfere with key signaling pathways such as NF-*κ*B, STAT3, and MAPK further underscores their therapeutic potential in pediatric psoriasis by attenuating keratinocyte hyperproliferation and inflammatory responses (38, 50). This integrated regulation of innate immune cell activity by natural products offers a promising strategy to modulate the cutaneous immune microenvironment effectively and safely.

### 3.2 Restoring skin barrier and microbiome homeostasis

**Figure 1:**
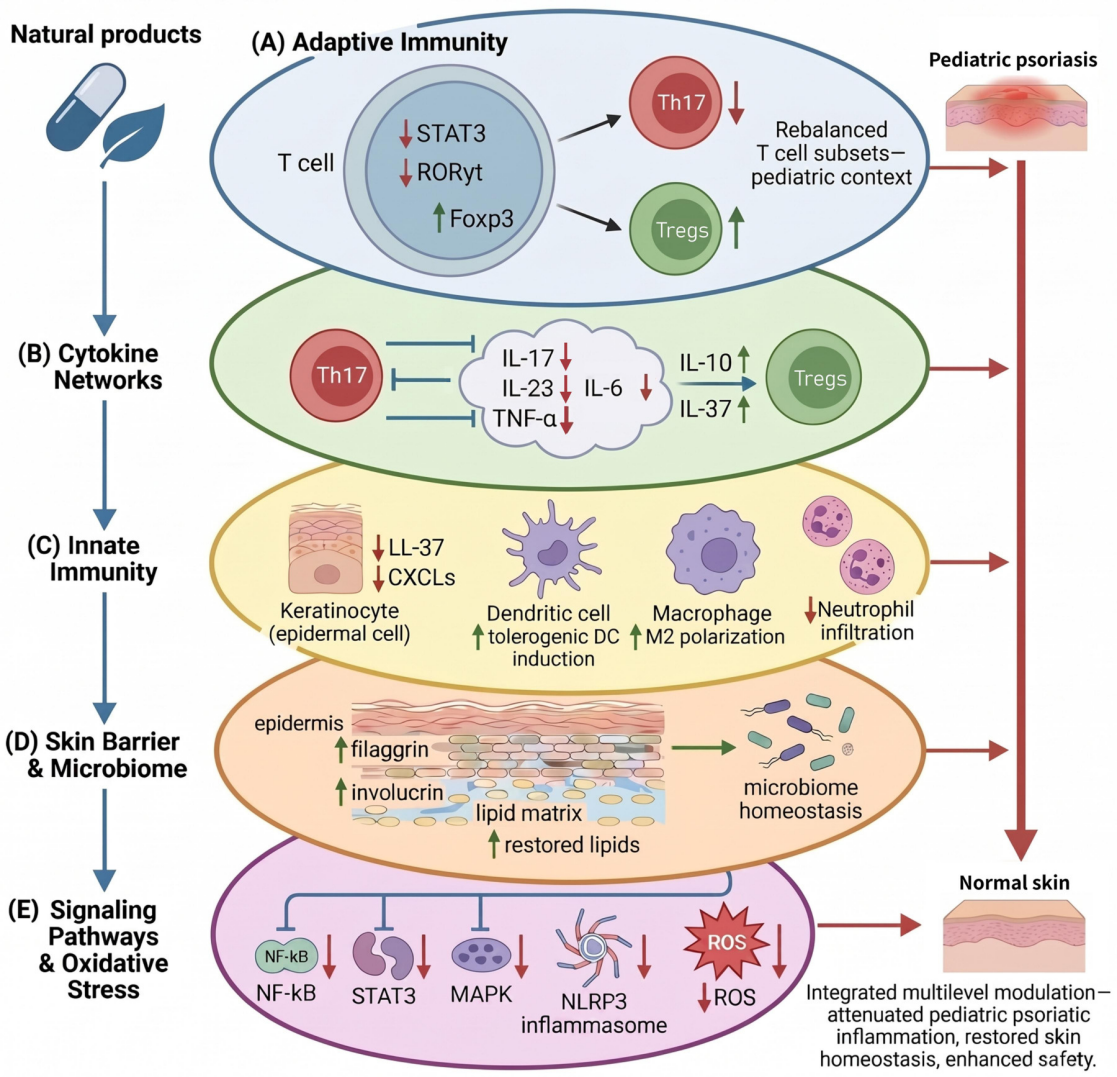
Multilevel immunomodulatory effects of natural products in pediatric psoriasis.

The integrity of the skin barrier and the balance of the cutaneous microbiome are fundamental to maintaining skin homeostasis and preventing inflammatory disorders such as psoriasis (51). Natural products contribute significantly to the restoration of the physical barrier by promoting keratinocyte differentiation and enhancing the expression of critical barrier proteins (52, 53). For instance, natural lipids including ceramide analogs and polyphenolic products have been shown to upregulate the synthesis of structural proteins such as filaggrin and involucrin, which are essential for the formation of the stratum corneum and its lipid matrix (54, 55). This upregulation facilitates the orderly arrangement of lipids in the stratum corneum, thereby reinforcing the skin's physical barrier and reducing transepidermal water loss. Moreover, the modulation of the skin microbiome by natural products is gaining recognition as an indirect yet vital mechanism for immune regulation. Certain natural products exhibit selective antimicrobial activity that suppresses the overgrowth of pathogenic bacteria such as *Staphylococcus aureus*, which is often implicated in exacerbating psoriatic inflammation. Simultaneously, these agents promote the colonization of beneficial commensal bacteria, thereby restoring microbial equilibrium and contributing to immune homeostasis (56). The skin-gut axis is particularly influential during childhood while the intestinal microbiota is still maturing, making children more susceptible to environmental triggers that disrupt this delicate balance. Natural products offer a unique advantage by not only neutralizing inflammatory signals but also stabilizing this axis, thereby preventing microbial metabolites from triggering systemic inflammation and restoring homeostatic balance within the cutaneous and intestinal microbiome (57). Oxidative stress is another critical factor that compromises skin barrier function and exacerbates inflammation (58). Natural antioxidants like tea polyphenols and resveratrol effectively scavenge excessive reactive oxygen species (ROS) in psoriatic lesions, mitigating oxidative damage to keratinocytes and immune cells (59). This antioxidant activity preserves cellular function and prevents apoptosis, further supporting barrier integrity and reducing inflammatory signaling (60). The combined effects of barrier protein enhancement, microbiome modulation, and antioxidative protection by natural products create a multifaceted defense against the pathogenesis of psoriasis (59, 61). These mechanisms not only repair the disrupted skin barrier but also recalibrate the local immune environment, highlighting the therapeutic significance of natural products in pediatric psoriasis management. The integration of these natural products into treatment regimens offers a promising avenue for restoring skin homeostasis with minimal adverse effects, addressing both the structural and immunological aspects of the disease. The multifaceted immunomodulatory actions of natural products are schematically illustrated in Figure 1, which integrates the adaptive, innate, and barrier-related effects described in Sections 2 and 3.

## 4 Translational applications and challenges in pediatric psoriasis

### 4.1 Developing pediatric treatment strategies

The development of pediatric treatment strategies for psoriasis leveraging natural products necessitates a nuanced approach tailored to the unique clinical and physiological characteristics of children. For mild and localized plaque psoriasis in children, topical formulations containing natural bioactive products such as glycyrrhizic acid and asiaticoside have emerged as promising first-line or adjunctive therapies (62–64). These agents, derived respectively from licorice root and *Centella asiatica*, exhibit anti-inflammatory and skin barrier-enhancing properties with favorable safety profiles, making them suitable for pediatric use where minimizing systemic exposure is paramount (65–67). The clinical viability of such botanical strategies is exemplified by TC cream, an extract of *Cnidium monnieri*, which recently received NDA approval following a Phase IIb trial that confirmed its safety and efficacy for treating psoriasis vulgaris (68). Their incorporation into topical ointments or creams, often combined with emollients, can effectively mitigate mild psoriatic lesions while reducing the risk of adverse effects commonly associated with corticosteroids or calcineurin inhibitors (4, 69, 70). In moderate to severe pediatric cases requiring systemic intervention, combining conventional systemic agents like methotrexate with natural hepatoprotective or immunomodulatory compounds such as silymarin (milk thistle extract) or tripterygium glycosides (from *Tripterygium wilfordii*) offers a potential strategy to enhance therapeutic efficacy and reduce toxicity (71, 72). However, such combinations demand rigorous clinical monitoring due to the complex pharmacodynamics and potential interactions inherent to natural products (73–75). Furthermore, a comparative perspective is essential: for instance, curcumin and resveratrol both demonstrate anti-inflammatory effects, but curcumin has more clinical data supporting its use in adult psoriasis, albeit with bioavailability challenges, while resveratrol's evidence remains largely preclinical. Glycyrrhizic acid stands out for its dual anti-inflammatory and barrier-restoring properties, making it particularly attractive for topical pediatric applications where safety is paramount. This comparative assessment highlights that agent selection should be guided by the strength of available evidence, the route of administration, and the specific clinical needs of children. Furthermore, the emerging concept of the gut-skin axis underscores the importance of systemic immunomodulation through modulation of the intestinal microbiome (76–78). Oral administration of probiotics or natural polysaccharide-based dietary supplements tailored to children may provide long-term benefits by restoring immune homeostasis and reducing psoriasis relapse rates (79). This holistic approach aligns with the immunoregulatory potential of natural products documented in various inflammatory skin diseases, emphasizing their role beyond mere symptom control to disease modification. Collectively, these strategies highlight the translational potential of natural products in pediatric psoriasis, balancing efficacy, safety, and the unique developmental considerations of children.

### 4.2 Clinical challenges and future perspectives

Despite the promising therapeutic potential of natural products in pediatric psoriasis, several critical challenges impede their clinical translation (15, 16). Foremost is the paucity of pediatric-specific formulations and standardized dosing regimens. Most existing research on natural products derives from adult populations or animal models, leaving a significant knowledge gap regarding pharmacokinetics, optimal dosing, and safety profiles across different pediatric age groups. The absence of palatable, age-appropriate dosage forms such as flavored oral liquids further limits adherence and therapeutic consistency in children. Equally critical are the unresolved issues surrounding product standardization, quality control, and dose consistency. The chemical composition of natural products can vary substantially depending on geographic origin, cultivation conditions, and extraction methods, leading to batch-to-batch variability that undermines both efficacy and safety. Without robust quality control frameworks akin to those applied to conventional pharmaceuticals, the reproducibility of therapeutic effects remains a major concern. Moreover, the potential for herb-drug interactions, particularly in children who may be receiving concomitant medications, has not been systematically studied and warrants explicit investigation. Additionally, the intrinsic complexity of natural products, often comprising multiple bioactive constituents, complicates quality control and mechanistic elucidation (17, 41). The synergistic or antagonistic interactions among these components pose significant hurdles for standardization and reproducibility of therapeutic effects. Advanced methodologies including network pharmacology and metabolomics are essential to systematically dissect these interactions and identify key active molecules responsible for immunomodulatory and anti-inflammatory actions. Another formidable barrier is the scarcity of high-quality evidence from large-scale, randomized, double-blind, placebo-controlled clinical trials specifically targeting pediatric psoriasis. This deficit undermines the establishment of robust efficacy and safety data necessary for regulatory approval and clinical guideline incorporation. Future research must prioritize well-designed clinical studies to generate reliable data supporting natural product use in children. Moreover, the integration of personalized medicine approaches holds promise for optimizing treatment outcomes. By leveraging genomic, immunophenotypic, and microbiome profiling, clinicians may identify pediatric subpopulations most likely to benefit from specific natural products, thereby enhancing precision and minimizing adverse effects. This precision approach aligns with the broader trend toward individualized therapy in dermatology and immunology. Looking ahead, the field should prioritize a transition toward targeted botanical drugs with clear molecular mechanisms, while leveraging AI-driven platforms and real-world evidence to design optimized combination therapies, such as integrating natural products with phototherapy or biologics, to further prolong remission periods in children (80, 81). In summary, overcoming these translational challenges requires concerted multidisciplinary efforts encompassing pharmaceutical development, rigorous clinical research, and precision medicine to fully harness the therapeutic potential of natural products in pediatric psoriasis. Table 2 outlines the current translational strategies and associated challenges for incorporating natural products into pediatric psoriasis management.

**Table 2:** Translational strategies and challenges of natural products in pediatric psoriasis.

Strategy	Examples	Potential Benefits	Challenges	Ref
Topical formulations	Glycyrrhizic acid, asiaticoside	Localized anti-inflammatory effect; minimal systemic exposure	Lack of pediatric-specific dosage forms; limited efficacy in moderate-to-severe cases	(62, 65, 66)
Systemic combination therapy	Silymarin + methotrexate	Enhanced efficacy; hepatoprotective effects	Risk of herb–drug interactions; need for pharmacokinetic studies in children	(71)
Oral immunomodulators	Probiotics, polysaccharide-based supplements	Gut–skin axis modulation; long-term immune homeostasis	Poor palatability; variable bioavailability; insufficient pediatric data	(42)
Microbiome-targeted interventions	Prebiotics, natural antimicrobials	Restores microbial balance; reduces *S. aureus* colonization	Limited clinical evidence in pediatric populations	(50, 78)
Personalized therapy	Genomic/ immunophenotypic profiling	Tailored treatment; improved safety and efficacy	High cost; need for validation in pediatric cohorts	(7, 35, 76)
Combination therapy	Co-administration of botanicals with phototherapy or biologics	Synergistic efficacy; prolonged remission; steroid-sparing effects	Uncertainty in drug-drug interactions; complex clinical management	(80)
Evidence-based digitalization	AI-driven drug screening, real-world evidence platforms	Accelerated lead discovery; personalized treatment optimization	High cost; requirement for multi-center data integration	(81)

## 5 Conclusion

The exploration of natural products in the management of pediatric psoriasis represents a promising yet nascent frontier, grounded in their multifaceted immunomodulatory effects. These agents exert influence through multiple targets and pathways, with preclinical evidence effectively restoring balance within the dysregulated immune microenvironment characteristic of psoriasis. By modulating T cell subsets, cytokine networks, innate immune cell functions, and reinforcing the skin barrier, natural products offer a comprehensive approach to disease intervention that aligns well with the complex pathophysiology of psoriasis.

From an expert perspective, the unique immunoregulatory properties of natural products position them as valuable candidates for pediatric psoriasis treatment, a domain where long-term safety is paramount. Their potential to serve as monotherapy, adjunctive therapy, or even as alternatives to conventional treatments addresses a critical need for safer, more tolerable options in children. This is particularly important given the chronic nature of psoriasis and the limitations associated with existing systemic therapies, which often carry risks of adverse effects and require careful monitoring.

However, the translation of these promising agents from bench to bedside in pediatric populations is not without significant challenges. Standardization of dosing regimens remains a fundamental hurdle, as variability in natural product composition can lead to inconsistent therapeutic outcomes. The development of pediatric-specific formulations is equally crucial to ensure appropriate pharmacokinetics, palatability, and adherence. Critically, issues of product quality control, batch-to-batch consistency, and the potential for herb-drug interactions must be systematically addressed before widespread clinical adoption can be considered. Moreover, a deeper mechanistic understanding of how these natural products interact with the pediatric immune system is essential to optimize their efficacy and safety profiles. This necessitates rigorous preclinical studies and well-designed clinical trials that specifically include children, rather than extrapolating data from adult populations.

Balancing the enthusiasm for natural products with the need for robust scientific validation requires a multidisciplinary approach. Integrating system biology techniques can unravel the complex interactions within the immune microenvironment and identify biomarkers predictive of response. Precision medicine frameworks can further tailor interventions to individual patient profiles, enhancing therapeutic outcomes while minimizing risks. Such strategies will facilitate the generation of high-quality clinical evidence, which is currently sparse but indispensable for regulatory approval and clinical guideline incorporation.

Looking forward, future research must prioritize translational studies that bridge laboratory findings with clinical application in pediatric psoriasis. Collaborative efforts among immunologists, pharmacologists, pediatric dermatologists, and bioinformaticians will be key to advancing this field. Emphasizing safety, efficacy, and personalization will not only improve disease management but also enhance quality of life for affected children and their families.

In conclusion, natural products hold significant promise as innovative, multi-targeted therapies for pediatric psoriasis, offering a potential paradigm shift in treatment strategies. However, realizing these potential hinges on overcoming critical challenges related to standardization, formulation, mechanistic insights, and evidence generation. By embracing integrative and precision medicine approaches, the field can move toward safe, effective, and individualized natural therapeutic options that address the unique needs of children with psoriasis. This balanced and rigorous pathway will ultimately translate into improved clinical outcomes and broaden the therapeutic arsenal available for this challenging chronic disease.
